# Supplementary material for: Risk factors for sacrococcygeal pilonidal sinus: a systematic review and meta-analysis supplemented by genetic causal assessment
Source: Front Surg. 2026 Jan 7;12:1718589. doi: 10.3389/fsurg.2025.1718589 (PMC12819706; doi:10.3389/fsurg.2025.1718589)
Supplement: Supplementary file 2 [file Datasheet2.zip › Supplementary Data 2/MR_pipeline_p5e-6/leaveone_plots/ebi-a-GCST90012030.finngen_R12_L12_PILONIDALCYST.pdf]

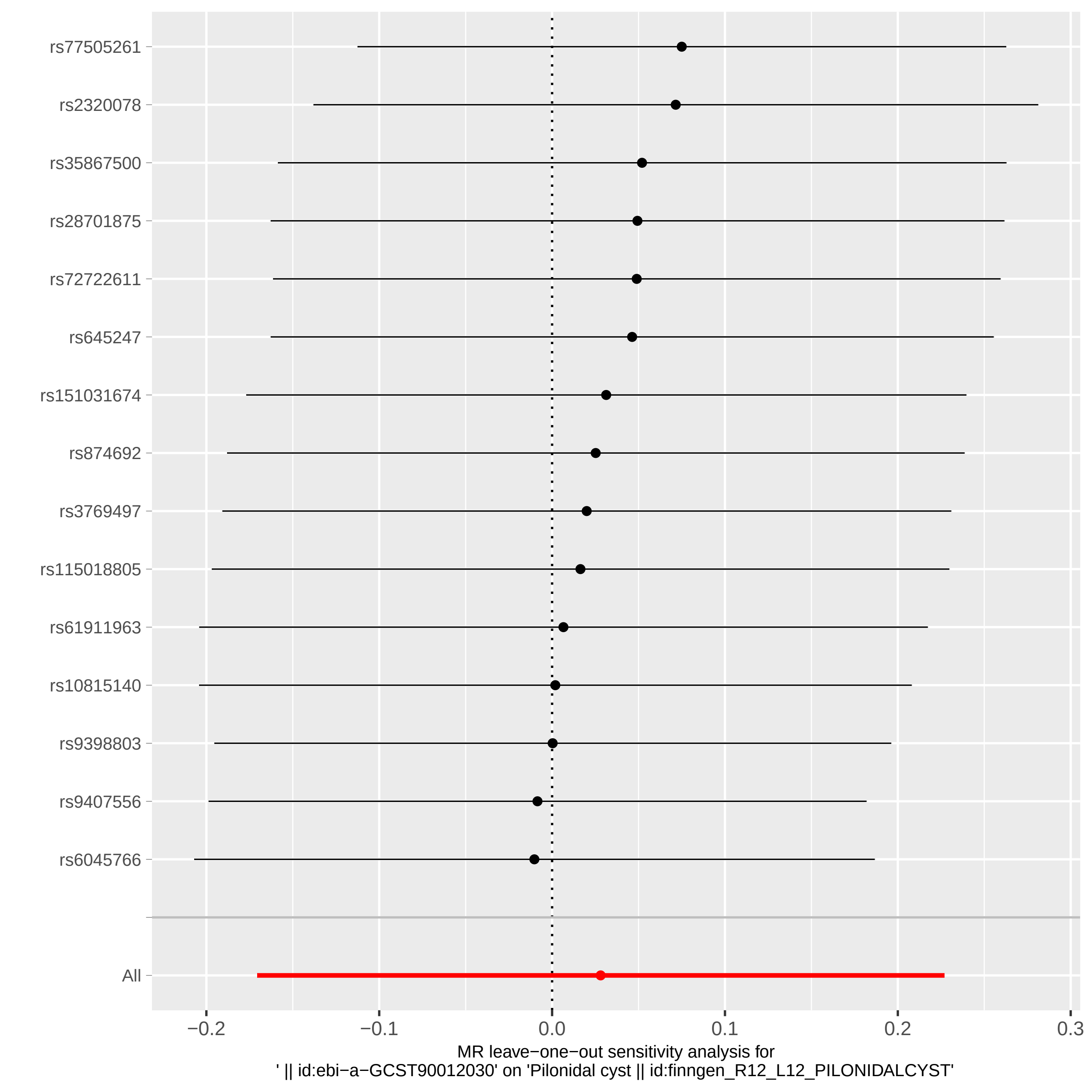
rs77505261
rs2320078
rs35867500
rs28701875
rs72722611
rs645247
rs151031674
rs874692
rs3769497
rs115018805
rs61911963
rs10815140
rs9398803
rs9407556
rs6045766
All
−0.2
−0.1
0.0
0.1
0.2
0.3
MR leave−one−out sensitivity analysis for
' || id:ebi−a−GCST90012030' on 'Pilonidal cyst || id:finngen_R12_L12_PILONIDALCYST'
